# Antecedents and Consequences of Grit Among Working Adults: A Transpersonal Psychology Perspective

**DOI:** 10.3389/fpsyg.2022.896231

**Published:** 2022-07-05

**Authors:** Devanshi Agrawal, Surekha Chukkali, Sabah Singh

**Affiliations:** ^1^Department of Psychology, Christ (Deemed to be University), Delhi, NCR, India; ^2^Department of Psychology, Panjab University, Chandigarh, India; ^3^Crayons Clinic, Chandigarh, India

**Keywords:** grit, transpersonal psychology, metacognition, flow, empathy, optimism, job satisfaction, job performance

## Abstract

Positive psychology has paved the way for newer and more informed ideas of living a meaningful, integrated and well-rounded quality of living. The current era of the pandemic has also moulded the ways in which individuals perceive their quality of life and how they want to integrate a holistic approach towards their well-being. The workplace settings have seen tremendous changes in terms of how employers, employees and the organisations at large function and operate. The pre-pandemic concept of success has shifted its focus from hard work to developing grit among employees to increase the overall efficiency of the organisations. Grit has revolutionised the conventional standards of success, meaning in life and has impacted personal as well as occupational welfare. This integration of positive psychology and transpersonal psychology has catalysed the purpose for the current study. To help organisations and individuals thrive in their professional endeavours at the workplace and to provide them with relevant psychological tools to enhance their occupational growth, the present study has been conducted empirically to investigate the antecedents and consequences of grit among 209 working professionals in India. The results of this study indicate that the transpersonal capital of metacognition, flow, optimism and empathy have a significant role to play in developing grit among the participants. The findings have implications for enhancing job satisfaction and job performance of participants. The current research also provides a framework to organisations towards designing interventions for improving efficiency at the workplace.

**JEL Classification code**: I10, I15, and I31

## Introduction

The phenomenon of grit has received considerable attention over the last decade. Grit, which has been defined as the passion and perseverance for long-term goals (Duckworth et al., 2007), is an essential predictor of long-term achievement and success. Research on grit in domains such as military, workplace sales, high school and marriage found that gritty individuals were less prone to withdrawing from their respective life commitments (Eskreis-Winkler et al., 2014). The year of 2021 is the year of the Great Resignation (Cook, 2021), wherein four million Americans quit their jobs in 2021 due to difficulty in managing work from home responsibilities, increased workload and burnout. The global pandemic has caused employees all over the world to rethink their life goals and whether it aligns with their current jobs. Lin et al. (2021) found that the COVID-19 was associated with job insecurity which was also related to emotional exhaustion and organisational deviance. Job satisfaction and organisational commitment were also found to be linked with each other during the global pandemic (Chanana, 2021). In light of this literature, it is important to help organisations retain their employees by assessing the factors that can contribute to increasing the well-being of employees. Grit is essential for organisations and employees to thrive (Lee and Duckworth, 2018). To explore the factors that predict grit and how grit in turn impacts employee job satisfaction and performance, a conceptual model is hypothesised to study the antecedents and consequences of grit among working adults in the present study.

In the ever-changing and fast paced world that has valued and emphasised the significance and implications of talent and hard work, which led to the achievement of success, as a major event that would occur in an individual’s life, has been widely prevalent (Mendick et al., 2015; Biondo and Rapisarda, 2018; Olivos, 2019). This meritocratic idea of possessing a conventional framework of personal and professional success, mostly extrinsically driven, has been challenged and has undergone a series of advancements in the way importance is ascribed to cultivation of meaning in life (Baczko-Dombi and Wysmulek, 2015). The reliance on innate talent was contradicted by Ericsson (2006) who coined the term “deliberate practice” to imply that continuous and repeated efforts over a period of time will lead to sustainable results and thereby assist in becoming an expert in a particular domain of career (Helding, 2011). Emphasis on environmental predictors of success for talent development has also found supporting evidence for creating an interactive space for personal and environmental factors to foster individual’s potentials in terms of developing persistence for making use of one’s talent (Subotnik et al., 2019). This shift from secluding the idea of success to talent and hard work, towards understanding the contextual psychological phenomenon involved in high performance, has led to the emergence of several theories of developing perseverance.

Revolutionised in the past decade, the emerging concept of grit, as a non-cognitive trait and a psychological tool to make sustained efforts towards achieving long-term goals in challenging domains, irrespective of talent (Duckworth et al., 2007) has received enormous recognition in the scientific community. Grit in the present paper can be understood from the framework given by Singh and Chukkali (2021) as the ability to adapt to situations, to show perseverance of effort, spirited initiative and steadfastness in not only long-term goals but also in situations requiring immediate attention. Grit provides contrary evidence to the conventional nature of possessing innate talents to obtain success. Measuring grit as a domain-specific construct in different achievement fields has found to be advantageous (Cormier et al., 2019). The emergence of grit in recent years has been tremendously rapid due to its utility and application in various settings such as healthcare, education and at the workplace (Christopoulou et al., 2018; Lee and Duckworth, 2018; Singh and Chopra, 2018; Musso et al., 2019). A systematic review by Fernández-Martín et al. (2020) revealed that grit serves as a predictor of educational, professional, personal success. In the educational setting, teaching grit can help students stay persistent and develop academic resilience in the face of adversity, self-regulate learning and achieve long-term challenging tasks and also prevents the depressive symptoms stimulated by positive and negative perfectionism (Hochanadel and Finamore, 2015; Ray and Brown, 2015; Bogin, 2017; Karlen et al., 2019; Xu et al., 2019; Tyumeneva et al., 2021; Yu et al., 2021a; Zhang et al., 2021; Chen et al., 2022). The pursuit of goals by being gritty is also linked with reduced chronic stress and lesser academic problems (Kalia, 2021) and associated with self-control but in a distinct form (Duckworth and Gross, 2014). In healthcare, possessing the qualities of grit helps healthcare workers perform better in crisis situations, assists in the quality of patient care being provided, can affect healthcare professionals’ job involvement and reduce burnout (Jeong et al., 2019; Tyer-Viola, 2019; Lee et al., 2021) The study of grit also exists in other dimensions of positive psychology. Due to the increased importance of enhancing meaning-making (Russo-Netzer, 2019), grit has been found to generate hope, meaning in life and 195 also leads to enhanced levels of flourishing (Vela et al., 2015; Valdez and Datu, 2021; Yang and Wu, 2021). Subjective well-being is also associated with grit (Kwon, 2021). Neural correlates of grit show prefrontal cortex and striatum and their functions that can contribute to individual differences in grit (Wang and Li, 2021).

Grit has significant implications during the COVID-19 pandemic in mitigating the effects of psychological distress imposed by the global health crisis. Research has found that grit can foster pandemic resilience to face the adversities caused by it (Bono et al., 2020; Schmahl, 2021). Individuals with higher levels of grit adopted better coping strategies leading to mitigated perceived stress by incorporating psychological tools such as minimisation and positive self-instruction during the COVID-19 lockdown and also results in inculcating healthier lifestyles to navigate the stressors of the global pandemic (Urban and Urban, 2020; Zepetnek et al., 2021). Interestingly, fear of the pandemic was also found to be safeguarded by higher levels of internal consistency, a principal component of grit, along with growth mindset, consequently reducing psychological distress (Masuyama et al., 2021; Mosanya, 2021). Pursuit of goals with grittiness is also connected to internal authenticity and possessing a sense of coherence (Vainio and Daukantaitė, 2016; Arya and Lal, 2018). On the other hand, grit has also been criticised on the grounds of not taking into account systemic privilege Schreiner (2017) as well as the decontextualized nature in which the concept of grit has been understood in the existing literature without any socio-cultural relevance and meaning provided for the same (Datu and Mclnerney, 2017; Kirchgasler, 2018). However, this concern was addressed by Morell et al. (2020) as it was found that the factor structures of grit scales such as perseverance of effort in the Grit-S (Duckworth and Quinn, 2009) was revealed to be the strongest determinant across three different samples and hence, provided evidence for how grit is not consistent across ages and cultures but can be significant in particular contexts. Therefore, it can be appropriately stated that the existing literature evidently highlights the relevance and usefulness of grit for individuals across the eastern and the western parts of the world, and grit predominantly increases psychological well-being across different career domains and professions (Salles et al., 2014; Jin and Kim, 2017; Arya and Lal, 2018; Datu, 2021).

It is imperative to look at the various concepts laid down by researchers investigating the psychology of achievement from the western and eastern perspectives. One of the pioneering researchers in this area, Dweck (2019) coined the term “growth mindset” referring to the ability to develop the belief that intellectual abilities can be learnt and fostered over time which is different from a “fixed mindset” wherein people believe that abilities cannot be learnt and are fixed. The application of the growth mindset has especially provided assistance to academic enhancement and improvement in student learning outcomes (Hochanadel and Finamore, 2015; Claro et al., 2016; Yeager et al., 2016; McCabe et al., 2020; Chen et al., 2022). The triarchic model of grit conceptualised by Datu et al. (2017) brought forth a newer framework for assessing grit, with the additional dimension of adaptability to situations to the existing two factor theory of grit (Duckworth et al., 2007) consisting of perseverance of efforts and consistency in interests. This triarchic model was linked with career exploration and talent development. Armstrong et al. (2018) developed a new model of grit in the context of self-regulation which found six strategies namely, temporal perspective, perpetual evaluation, motivational orientation, strength and resource gathering, system thinking and framing, that can be used by organisations to inculcate grittiness among their leaders of innovation.

Jachimowicz et al. (2018) defined passion as a strong feeling towards an important value or preference in individuals and found that previous studies had only emphasised perseverance and not on passion and, thus, indicated that the amalgamation of perseverance and passion can significantly benefit the development of grit. Another framework for assessing workplace goal orientation was assessed by developing a goal orientation process model namely “GRRR” by Ceschi et al. (2021) which found grit as a predictor of resilience, in the form of construct relationships of grit leading to resilience which in turn, results in recovery, thus, proving to be a significant model in order to assess workplace long-term relationships among grit, resilience and recovery. To bridge the gap between grit researches in the eastern perspective, Singh and Chukkali (2021) developed a reliable framework for measuring grit, including dimensions of grit, adaptability to situation, perseverance of effort, spirited initiative and steadfastness in adverse situations. These perspectives provide deep and extensive insights into the advancement and implementation of grit in important areas of functioning in individual’s lives.

Drawing from the transpersonal psychology perspective, the present study is conducted for the purpose of taking the entire human experience into account. The field of transpersonal psychology, also known as the fourth force, was founded by Maslow who moved humanistic psychology into the spiritual realm (Hastings, 1999). The shift from humanistic psychology to transpersonal psychology took place to honour the entire spectrum of human experience by studying the intangible, but important parts of existence, mainly, spirituality and its transpersonal dimensions (Valle, 1989). Transpersonal phenomenon heralded to importance, to challenge the ethnocentric biases of the time (Grof, 2008). Transpersonal psychology is concerned with the “study of humanity’s highest potential, and with the recognition, understanding, and realisation of unitive, spiritual, and transcendent states of consciousness.” (Lajoie and Shapiro, 1992). It has been defined by Bynum (1992) as the study of psycho-spiritual disciplines and processes accounted for in the science and religion of the earliest discovered civilisations. The present paper draws the operational definition from the work of Hartelius et al. (2013) who explained transpersonal psychology as “a transformative psychology of the whole person in an intimate relationship with an interconnected and evolving world, it pays special attention to self-expansive states as well as to spiritual, mystical, and other exceptional human experiences that gain meaning in such a context.” Daniels (2013) identified four distinct perspectives within transpersonal psychology, namely, religious, psychological, humanistic/existential/feminist and ecological perspectives. The transcendent and reflective nature of this field has recognition due to its applicability and significance, over the past few decades and is pivotal to human survival and advancement in life (Friedman, 2002; Ardelt and Grunwald, 2018). To expound on the broader meaning of daily work and living, Adams (2019) captures the essence of how the transpersonal quality is deeply embedded in our personal and vocational domains, thus, studying existential concerns through phenomenology and contemplative spirituality. While considerable attention has been given to the more widespread forms of transcendence, such as meditation, prayer, non-ordinary states of consciousness, transcendence can also found within daily life experiences and in simple events of interpersonal relationships, in the openness to gather knowledge of different cultures, environmental activism and even in contemporary physics (Clements et al., 2016). Often misconstrued to be inclusive of supernatural speculations, transpersonal psychology is instead, a science that helps in understanding the interconnectedness individuals can feel, when they identify with their external environments, their past, present and future and are engaged in expanding the traditional and conventional ways in which life should be viewed, lived and perceived (Friedman, 2018). Grit and transpersonal psychology are linked with each other through their deeper constructs and mechanisms. Spirituality, an important component of transpersonal psychology (Hartelius et al., 2013), has a positive relationship with grit (Dutta and Singh, 2017). Flow, the antecedent of grit, as discussed in the present paper, is facilitated through self-transcendence (Osin et al., 2016) and serves as a transpersonal transformative practice for individuals (Galloway, 2005).

Employee well-being since the inception of the COVID-19 pandemic has compelled organisations to introduce and implement new solutions to tackle ordeals and challenges across myriad areas of operations among the abrupt change in working conditions (Carnevale and Hatak, 2020; Diab-Bahman and Al-Enzi, 2020). The pandemic heralded a new era of remote work along with many other unique and unprecedented challenges affecting the well-being as well as organisational functioning of workplaces. Evidently, it was found by Juchnowicz and Kinowska (2021) that being involved in remote work had a negative impact on affect well-being in terms of work-life balance and relationships. Research also indicates that stress induced due to the COVID-19 pandemic led to reduced employee performance (Yu et al., 2021b). Occupational health became a central area of study during the pandemic due to the tremendous employee exhaustion, job insecurity, organisational layoffs, decreased social support which were some of the major factors in impacting employee well-being during the pandemic (Kriz et al., 2021; Meyer et al., 2021). Firms with employee satisfaction have also been found to navigate the crisis of the pandemic more efficiently (Shan and Tang, 2020). Several employee engagement practices have also been made use of, to boost and increase motivation among employees to lead to professional and personal development in the form of recognition and acknowledgment sessions, e-learning modules and work-from-home regime tasks and activities which led to increase in employee commitment towards the organisation (Chanana and Sangeeta, 2020). Other than these practices, the presence of a supportive work environment comprising interpersonal trust in colleagues and managers can lead to job satisfaction (Bulińska-Stangrecka and Bagieńska, 2021). Interestingly, Nemteanu and Dabija (2021) reported that an emerging area such as internal marketing, which implies promoting the values and objectives of the organisation towards the employees themselves, also has a positive impact on job satisfaction.

Grit at the workplace is an under researched area since most of the literature till date has explored grit in the educational setting. Lee and Duckworth (2018) found that to help organisations obtain a culture of grit, it is imperative to select individuals who are gritty. Evidently, the existing literature provides findings for utilising the concept of grit to hire employees and to assign meaningful work to foster their engagement (Singh and Chopra, 2018) but is also useful for job-seekers in optimising job search performance (van der Vaart et al., 2021). Grit is also considered worthy of practical application by employers in the hiring process and deemed more than just a theoretical concept (Butz et al., 2019). Grit also improves the organisational environment of the workplace in specific constructs such as strengthening stamina and resilience of employees (de Waal et al., 2022). Choi et al. (2020) found that grit not only strengthens the relationship between corporate social responsibility and meaning orientation but also leads to an improved organisational citizenship behaviour. Since workplace politics also plays a significant role in the functioning of an organisation, grit, both in employees and supervisors, neutralises the political dynamics of the workplace at hand (Jordan et al., 2018a,b). Similar results of the significance of grit at the workplace were found by Popoola and Karadas (2022) as the presence of grit was associated with career success. Partially supporting these findings, Clark and Plano Clark (2019) found that grit is necessary for career success but other factors such as luck, networking and opportunity play an an important role in determining success in one’s career and in the Asian context, components of personal motivation, social support system must be considered while conceptualising grit (Vera et al., 2015). The findings of these studies are imperative of the fact that grit serves as an essential psychological tool while navigating the ecosystem of workplaces.

Grit, in the isolation of passion and perseverance in promoting well-being of individuals has been challenged and called into question as not being psychometrically unsatisfactory (Credé, 2018; Tynan, 2021). Existing literature on employee well-being during the COVID-19 pandemic has profoundly benefited organisations but lacks the approach of taking the entire human experience into account and is also inadequate in providing a dynamic and broad conceptual model on dealing with the workplace challenges brought about by the inception of the COVID-19 pandemic from a holistic perspective. Additionally, previous research is insufficient in terms of recognising the need, importance and implementation for fostering grit among working professionals by inculcating transpersonal factors such as metacognition, flow, empathy and optimism. Current knowledge on predictors has found hope and low rumination as the antecedents of grit (Raphiphatthana and Jose, 2021) which, although significantly helps in advancing research on constructs of positive psychology in relation to grit, but is limited in providing a sustainable framework for developing and generating grit, especially at workplaces.

Olckers and Koekemoer (2021) explored the underlying mechanisms which can contribute to career success and found psychological ownership as the possible construct influencing performance through grit and the perception of career success. Research also states that grit is mainly useful in the presence of socioeconomic resources and individuals with higher incomes are more likely to become entrepreneurs (Arco-Tirado et al., 2019). Hill et al. (2016) also examined the predictors of grit and found life direction as a predictor of becoming grittier. However, the existing body of research has not yielded beneficial results in developing a model that could contextualise an individual’s socioeconomic and other concerns by helping them inculcate qualities of transpersonal capital that could significantly improve their overall well-being as well as enhance their job satisfaction and job performance. There is a need for examining grit beyond the two factor theory of passion and perseverance (Datu, 2021).

There is a gap in the present literature in terms of giving a deep understanding and insight into the underlying mechanisms of grit and how grit can enhance the occupational health of employees and employers. Thus, the present study aims to fill this gap by providing an eastern perspective of the antecedents and consequences of grit. The current paper tackles these issues through its formulation of a conceptual framework that could help organisations thrive and foster employee satisfaction and performance through the perspective of transpersonal psychology.

In the context of the current study, the transpersonal capital of metacognition, optimism, flow and empathy are of utmost significance and importance to workplaces, owing to their inclusive and comprehensive nature of providing a deep understanding and insight into expanding the well-being of individuals. There is supporting evidence to the expansiveness of the transpersonal perspective suggesting that self-transcendence can be facilitative of meaning-making and flow experiences and these subjective flow encounters also mediate psychological capital and happiness at work (Osin et al., 2016; Kawalya et al., 2019). To increase workplace spirituality, Palframan and Lancaster (2019) drew their findings from the transpersonal model and observed that self-reconciliation can lead to meaning-making and enhancement of self-expression and inner purpose at the workplace. Usman et al. (2020) also suggest and recommend the usage of transpersonal perspectives such as Sufism, to improve well-being and mental health at the workplace and to deal with workplace stressors and anxiety. The study of values and transformation to enhance workplace spirituality has been of recent focus and has been receiving importance due to its beneficial implications for organisations (Kochukalam et al., 2018; Palframan and Lancaster, 2019; Dubey et al., 2020; Khari and Sinha, 2020).

The usage of the transpersonal psychology perspective has not been implemented or thoroughly researched earlier in terms of enhancing workplace management (Avramchuk, 2020). Through this theoretical perspective of transpersonal psychology, the present study aims to explore the relationship between the antecedents and consequences of grit at the workplace among working professionals in India from the perspective of transpersonal psychology.

## Materials and Methods

The study followed a quantitative approach for data collection and analysis. The sample was selected on the basis of inclusion and exclusion criteria. Purposive sampling was used to selectively obtain data from working professionals living in India along with other criteria for selection for participation in the study. All measures were in the English language.

### Participants

The data were collected from 209 participants who were working professionals in organisations in India through the online platform of Google Forms (Vasantha Raju and Harinarayana, 2016; Torrentira, 2020) which consisted of questionnaires of the variables being measured in the study, namely, grit, metacognition, optimism, flow, empathy, job satisfaction and job performance. Purposive sampling method was used to gather participants for data collection. The informed consent of the participants was taken before beginning the data collection as part of an important ethical consideration to respect human rights (Mumford, 2018). They were informed about the purpose of the study and about the benefits of participating in the research as mentioned in the form itself for their voluntary participation. The inclusion criteria of the sample were as follows: (a) working professionals in an organisation in India; (b) are citizens of India; (c) are above the age of 18 years. The responses of participants who did not meet the inclusion criteria were eliminated. Table 1 demonstrates the descriptive statistics reported in the study in the data collection.

**Table 1:** Descriptive statistics of participants.

Variable		Frequency	Percentage (%)	Mean	SD
Age	20–63			26.04	6.087
Gender	Male	117	56		
	Female	92	44		
Educational qualification	Post-graduation	65	31.1		
	Graduation	149	67		
	Diploma	4	1.9		
Home town	Urban	187	89.5		
	Rural	22	10.5		

### Measures

#### Grit

Grit was assessed using the Multidimensional Scale of Grit developed by Singh and Chukkali (2021) which measures four factors of grit, namely adaptability to situation, perseverance of effort, spirited initiative, steadfastness in adverse situations. It is a 12-item scale. There is no reverse scoring and the minimum score is 12 and maximum is 60. The scale has a good reliability of 0.795. Convergent validity indicated positive correlation with PCASS (*r* = 0.527) and Revised Norwegian Dispositional Resilience (Hardiness) Scale (*r* = 0.565).

#### Metacognition

Metacognition was assessed using the Metacognition Self-Assessment Scale developed by Pedone et al. (2017) which measures five abilities of metacognition, which are monitoring, differentiation, integration, decentration and mastery. It is scored on a 5-point Likert scale with a minimum score of 18 and a maximum score of 90. To measure reliability, Cronbach’s alpha ranged from 0.72 and 0.87. The MSAS has a good factorial validity and internal consistency.

#### Optimism

Optimism was assessed using the Life Orientation Test-Revised developed by Scheier et al. (1994) and is used to measure optimism versus pessimism. It is a 10-item 4-point Likert scale and consists of reverse scoring of items 3, 7 and 9. The sum total of items 1,3, 4, 7, 9 and 10 is taken for the individual scores of participants. Cronbach’s alpha was found to be 0.78 indicating adequate internal consistency. The convergent and discriminant validity of the LOT-R are also acceptable.

#### Flow

Flow was assessed using the Flow Short Scale developed by Rheinberg et al. (2003) and cf. Engeser and Rheinberg (2008) and is a 13-item 7-point Likert scale measuring flow and worry. The English version of the scale (Rheinberg, 2015) was used for measuring flow. Items measuring flow were summed for the use of the present research. Cronbach’s alpha ranged between 0.80 and 0.90. Administration of the Flow Short Scale takes 30–45 s.

#### Empathy

Empathy was assessed using the Toronto Empathy Questionnaire developed by Spreng et al. (2009) and is a 16-item questionnaire with strong convergent validity, internal consistency and high test–retest reliability. The TEQ consists of negatively worded items for reverse scoring that are items 2, 4, 7, 10, 11, 12, 14 and 15. The sum of all the scores is used for derivation of total scores.

#### Job Satisfaction

Job satisfaction was assessed using the Job Satisfaction Survey developed by Spector (1985) which is a 36-item survey ranging from “strongly disagree” to “strongly agree” measuring nine sub-scales namely, pay, promotion, supervision, fringe benefits, contingent rewards, operating conditions, coworkers, nature of work, communication and the total satisfaction is calculated by the sum of all the 36 items. The survey also consists of negatively worded items which are 2, 4, 6, 8, 10, 12, 14, 16, 18, 19, 21, 23, 24, 26, 29, 31, 32, 34 and 36. High scores obtained on the scale indicate job satisfaction. The psychometric properties of the survey indicate high internal consistency reliability of 0.91 and is a widely used measure of job satisfaction.

#### Job Performance

Job performance was assessed using the Individual Work Performance Questionnaire developed by Koopmans et al. (2013) which measures three dimensions of work performance, namely, task performance, contextual performance and counterproductive work behaviour. It is an 18-item questionnaire and the scores for the three dimensions are obtained separately. The internal consistency, convergent validity and discriminative validity of the IWPQ are acceptable. It is widely used for research purposes in assessing work performance and serves as a reliable and valid instrument for examining individual work performance in different occupational sectors (Koopmans et al., 2014).

#### Procedure

The Institutional Ethics Committee reviewed the application for the present research and gave the necessary permissions for the ethical clearance for data collection. A pilot study was conducted with 30 respondents to estimate the time taken and the relevance of responses received through the tools being used for the study. After the successful response in the pilot study, the data were then collected for the full-scale study. The informed consent for voluntary participation was collected from the participants before the conduction of the study. The data collection was conducted on an online platform with the inclusion of all the above-mentioned measures in a self-reported form (Mutepfa and Tapera, 2018). The items of the measures were listed in a sequential form, in the following manner, starting from the Multidimensional Scale of Grit, Metacognition Self-Assessment Scale, Flow Short Scale, Life Orientation Test-Revised, Toronto Empathy Questionnaire, Job Satisfaction Survey and Individual Work Performance Questionnaire. The administration of the test included giving a brief introduction of the study as well as its nature, purpose and use in the academic research. The participants were also informed about their rights of confidentiality, privacy, anonymity and withdrawal in order to make an informed decision to participate in the study (Allen, 2017).

### Data Analysis

#### G*Power

To estimate the required sample size for the study, the G* Power 3.1 software was used, which is a widely used software for determining sample size and to conduct power analysis (Faul et al., 2009; Kang, 2021). The results from the Power Analysis on G-Power revealed that the minimum sample size in the study should be 138, which has been achieved.

#### Harman’s Single Factor Test

To assess the common method bias which refers to the presence of systematic variance in the undertaken measures, which can have an impact on the reliability and validity of the tools employed in the research (Aguirre-Urreta and Hu, 2019), the Harman’s Single Factor Test has been employed on SPSS Statistics 28.0.1. To conduct this test, one single factor is extracted by loading all the items into one common factor. The total variance for the common factor was found to be 24%, which is less than 50%, indicating that there is no presence of common method bias in the measures.

Statistical analyses were performed using the following software and statistical methods. The total scores of all measures were used to conduct the analysis to ensure uniformity. To substantiate the relationship between the antecedents (metacognition, optimism, flow, empathy) and consequences (job satisfaction, task performance, contextual performance and counterproductive work behaviour) of grit, the Pearson correlation analysis was conducted on IBM SPSS Statistics 26.0 (Antonius, 2012). Furthermore, to test the hypothesised associations among the exogenous and endogenous variables based on the proposed conceptual framework, IBM SPSS Amos 27.0 was used (Arbuckle, 2011)

## Results

A total of 209 working professionals living in India in the age range of 20–63 years, males (56%) and females (44%) working at organisations in different sectors were a part of the study. 31.1% were post-graduates, 67% were undergraduates and 1.7% reported to have done diplomas. Also, it was noted that 89.5% belonged to urban areas, whereas 10.5% belonged to rural areas, as shown in Table 1. To measure for sample adequacy, an exploratory factor analysis was performed and the KMO (Kaiser–Meyer–Olkin) test was performed and was found to be 0.749 which represents acceptable sample adequacy and the results of Bartlett’s Test of Sphericity revealed the correlation matrix to not be random *χ*^2^ (36) = 588.862, *p* = <0.001, and was found suitable for the factor analysis to be performed (Singh and Chukkali, 2021).

Table 2 shows the correlation among the variables measured in the study. Pearson correlation analysis found that grit is positively correlated with metacognition (*r* = 0.662, *p* < 0.01), optimism (*r* = 0.162, *p* < 0.05), flow (*r* = 0.162, *p* < 0.05), job satisfaction (*r* = 0.173, *p* < 0.05), task performance (*r* = 0.441, *p* < 0.01) and contextual performance (*r* = 0.565, *p* < 0.01).

**Table 2:** Correlation analysis among study variables.

Variable	Mean	SD	(1)	(2)	(3)	(4)	(5)	(6)	(7)	(8)
Grit	49.30	7.03	0.622**	0.162*	0.537**	0.002	0.173*	0.441**	0.565**	−0.042

(1) Metacognition, (2) optimism, (3) flow, (4) empathy, (5) job satisfaction, (6) task performance, (7) contextual performance, and (8) counterproductive work behaviour. SD, standard deviation.

** *p* < 0.01;

* *p* < 0.05.

**Figure 1:**
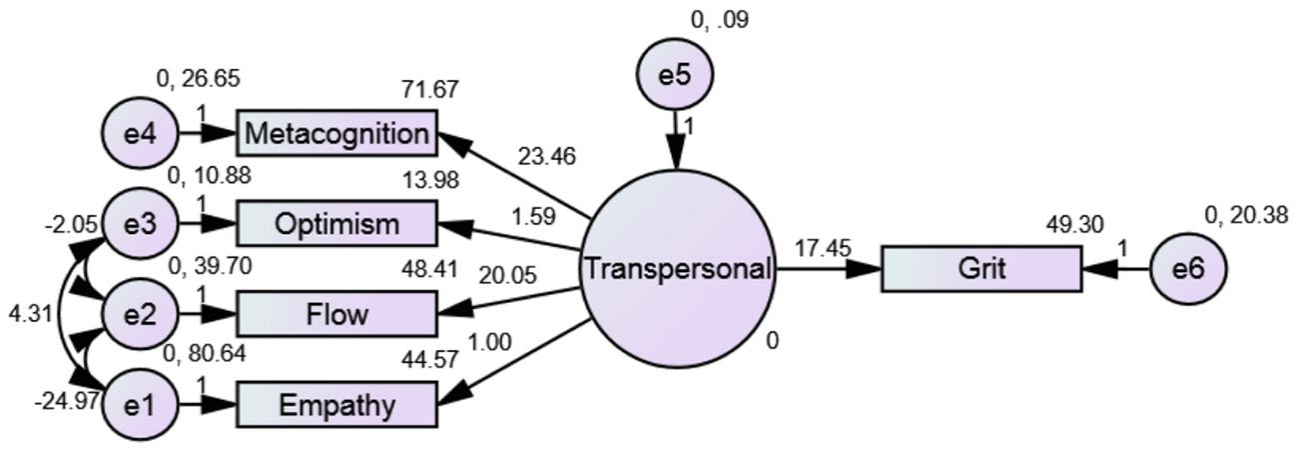
Confirmatory factor analysis of antecedents of grit.

**Figure 2:**
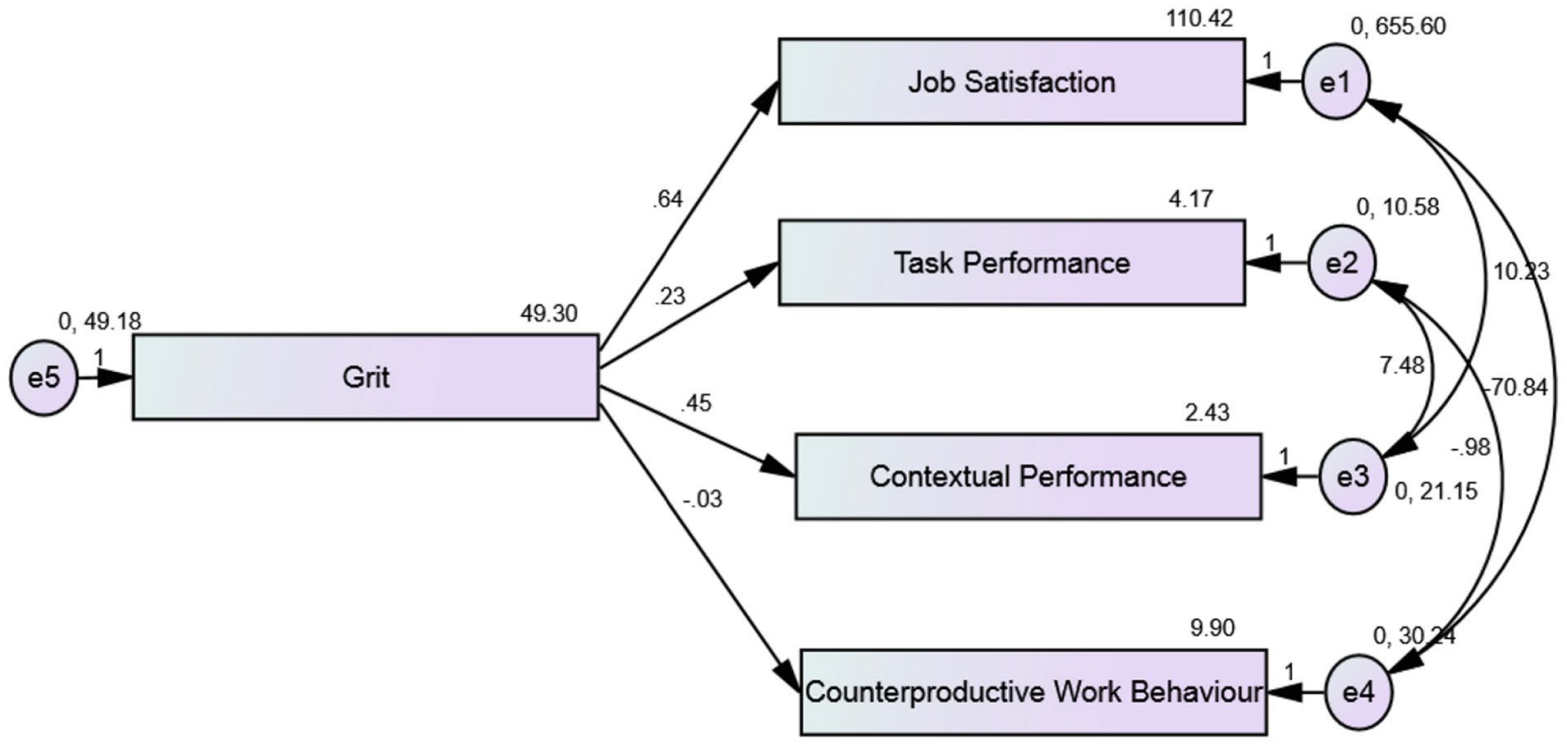
Confirmatory factor analysis of consequences of grit.

Two confirmatory factor analyses were performed, one for the antecedents of grit and one for consequences of grit to ensure that the measurement models were adequate. The model fitted the data acceptably for antecedents of grit, *χ*^2^ = 3.144, *p* = 0.208, CFI = 0.995, RMSEA = 0.052, SRMR = 0.018, NFI = 0.987, TLI = 0.976, AIC = 39.144, and for consequences of grit *χ*^2^ = 2.740, *p* = 0.254, CFI = 0.997, RMSEA = 0.042, SRMR = 0.035, NFI = 0.990, TLI = 0.985, AIC = 38.740, respectively, and indicate a good fit for both the models (Schumacker and Lomax, 2010; Figures 1, 2; Tables 3, 4).

**Table 3:** Fit indices of the CFA model of antecedents of grit.

Model	*df*	Chi square	RMSEA	CFI	NFI	SRMR	TLI	AIC	*p*
Default model	2	3.144	0.052	0.995	0.987	0.018	0.976	39.144	0.208

**Table 4:** Fit indices of the CFA model of consequences of grit.

Model	*df*	Chi square	RMSEA	CFI	NFI	SRMR	TLI	AIC	*p*
Default model	2	2.740	0.042	0.997	0.990	0.035	0.985	38.740	0.254

## Discussion

The present study aimed to examine the multiple causal relationship between the antecedents and the consequences of grit. To bridge the gap between the need for a conceptual model for exploring the factors contributing to grit and its outcomes to help organisational sectors thrive, in the context of an eastern perspective, the theoretical perspective of transpersonal psychology, the hypothesised conceptual framework was formed, with the transpersonal factors of metacognition, optimism, flow, empathy as predictors of grit and job satisfaction and job performance (task performance, contextual performance and counterproductive work behaviour) as consequences of grit. Based on the data collected from 209 working professionals living in India, working in different parts of India, the findings of the analyses were found to be consistent with the hypothesis of the study that transpersonal capital predicts grit which, in turn, predicts job satisfaction and job performance. Grit was strongly associated with metacognition, optimism, job satisfaction and task performance, consistent with the existing body of literature (Arslan et al., 2013; Min Hee and Sook Hee, 2019; Chandrawaty and Widodo, 2020; Clement et al., 2020).

The relevance of the findings of the present study, implicating that the transpersonal factors of metacognition, flow, empathy and optimism can result in grit, can be due to the acknowledgement and inclusion of the contemporary human experience resulting from transpersonal psychology (Hartelius et al., 2007). The results are also consistent with the current knowledge on the antecedents of grit, as flow experiences at the workplace have been found to be effective tools for reconstructing individual and team performance (Aubé et al., 2014; Hout et al., 2018) as well as in fostering well-being (Peifer et al., 2020) and job satisfaction (Maeran and Cangiano, 2013). The positive association between grit and metacognition found in the present research can be attributed to the metacognitive processes in which individuals and teams engage in, during crucial decision making tasks (McCarthy and Garavan, 2008). Metacognition has been studied in conjunction with theory of mind, social cognition and self-regulation (Baker et al., 2020) and it has also been identified important to study job-seeking behaviours and job-search outcomes, as the process of seeking and finding jobs requires metacognitive skills, implying the ability to set goals, plan, develop and strategise their job search progress and job performance (Mikulecky and Ehlinger, 1986; Turban et al., 2009; Kanar and Bouckenooghe, 2021). The other transpersonal capital of optimism also positively correlates with grit, consistent with previous studies (Steinfort, 2015; Clement et al., 2020; Loftus et al., 2020; Oriol et al., 2020; Kim and Lee, 2021). Optimism has also been shown to foster and predict perseverance, an essential component of grit (Binsch et al., 2017). Congruent with the results of the present study, utilisation of positive psychology based workplace training programmes to stimulate a culture of optimism in organisations to promote inculcation of positive emotions is also an advantageous method to advance a sense of well-being at the workplace (Pykett and Enright, 2016). Similar findings were found by Malik (2013) who indicated that optimism can help maintain positive organisational behaviour at the workplace. The positive association between grit and empathy in the present research is consistent with the existing literature which suggests that higher levels of grit indicate higher empathic orientation (Isenberg et al., 2020) and Singh (2014) who established that fostering empathy at the workplace can help in adapting to different situations. Interestingly, Paakkanen et al. (2021) stated that validating and constructive responses can help in developing positive emotions among coworkers.

The field of transpersonal psychology has seen a shift from its emphasis on altered states of consciousness towards an inclusive, diverse, holistic, experience of simple transcendence at the individual and collective levels (Hartelius et al., 2021; Richards Crouch et al., 2021). Transpersonal psychology is one of the only areas in the discipline of psychology encompassing the wide dimensions of spirituality, which focuses on achieving the epitome of human potential (Cowley, 1993). This perspective in the current study offers interesting insights into the occupational health of employees. The pandemic saw a considerable decline in work satisfaction among employees and the indirect effects of social isolation on remote work satisfaction (Toscano and Zappalà, 2020; Möhring et al., 2021) as well as the impact on the well-being of employees due to financial situation and physical health (Harju et al., 2021), making it crucial to identify the need to address the health emergency that has arisen due to the deadly pandemic. In this context, incorporating the perspective of transpersonal psychology into inculcating and improving the mental, physical, emotional and overall well-being of the working professionals community is of utmost importance. Law and Buckler (2021) substantiated the usefulness of transpersonal coaching in times of the COVID-19 pandemic to reconstructing new meaning on the basis of the past, to expand one’s sense of being, to experience the higher self, to reach a state of self-transcendence, and to integrate mindfulness in one’s life, thereby positively impacting our intrapersonal and interpersonal relationships, our environment and the planet at large. The role of self-transcendence, which implies reaching beyond one’s immediate confines and inculcating the quality which may result due to this process, which has been found as a measurable construct of transpersonal psychology (Garcia-Romeu, 2010), has become an crucial state for seeking relief and dealing with uncertainty during the pandemic (Worth and Smith, 2021). Other studies support the importance behind this construct, by recognising how meaning-making and self-transcendence can affect well-being, facilitate healing and serve as a buffer against suffering imposed by the COVID-19 pandemic (Wong, 2016; Wong et al., 2021). Henceforth, it can be stated that the entirety of human experience that transpersonal psychology seeks to promote and facilitate, can help individuals deal with the inevitable suffering caused by the pandemic, emotionally, physically and on a deep, collective level.

The necessity of a framework providing insights into understanding how to inculcate grit within working professionals and how grit in itself can influence and enhance job satisfaction and job performance at the workplace has played a pivotal role in the foundation of the present study. The need for grit to be integrated within a conceptualised framework has been ongoing in recent times (Jordan et al., 2019; Kim et al., 2021; Sudina and Plonsky, 2021; de Waal et al., 2022) but has left a gap in addressing the well-being and occupational health of employees from the lens of transpersonal psychology. Previous research on conceptualising grit include an attempt by Southwick et al. (2019) to provide the organisational antecedents of grit, namely leadership, culture and job design with the consequences of employee retention, engagement and job performance. The present research has furthered the work of the former researchers on grit, by acknowledging employee welfare from a broader lens by focusing attention on metacognitive, flow experiences and inculcating empathy and optimism, to sustain the pressures of life. The work by (Claro et al., 2016; Fitzgerald, 2016; Yeager et al., 2019; Wolcott et al., 2020) on growth mindset which implies that mindset is a construct that can permeate across socioeconomic disadvantages, is facilitative of the results of the current study, as it means that the non-cognitive trait can be learnt through development of metacognition, flow, optimism and empathy within working professionals, with the collective support of a positive organisational culture, thereby also contributing to transformational leadership at work (Caniëls et al., 2018).

The implications of this study are several, including the potential for the conceptual framework of antecedents and consequences of grit from a transpersonal psychology perspective to be utilised, studied and implemented in organisations to improve employee well-being, health, welfare and to improve their levels of satisfaction and performance. Since the study is one of the pioneering researchers in understanding occupational outcomes from a transpersonal lens, it can serve as a guiding foundation for nurturing the capabilities and strengths of employees, considering that the study is one of the first in offering an eastern perspective of the factors behind the development of grit and its outcomes, in the context of the collectivist groundwork of India. Companies, organisations and working professionals across India have faced the setbacks of the transition from shifting from offline to remote work as well as occupational layoffs, burnout, decrease in work performance, work engagement, job satisfaction with a reduction in personal well-being. The inclusion and integration of grit through the transpersonal capital of metacognition, flow, optimism, empathy can significantly elevate not only the performance and satisfaction of employees, but will also equip themselves with psychological tools to help them navigate the ordeals and uncertainties of everyday life and henceforth, it will create a sense of meaning towards their work and personal life.

There are a few limitations in the study. Firstly, due to the online collection of data, there is room for error and inaccurate responses by participants owing to technical difficulties. Owing to the pandemic, the data could not be collected in person, thus, increasing the level of portraying oneself socially desirable on the online data collection platform. Secondly, the constructs being measured in the study, are not a part of everyday occupational vocabulary, thereby, making it important for organisations to truly understand the constructs before trying to inculcate, improve and implement them in their workplace settings, especially in rural workplace settings which may not be familiar with these constructs, as majority of the participants in the study belonged to urban settings. Hence, further efforts can be made to inform and elevate the understanding of organisations for improving efficiency among employees. Thirdly, more diverse research can be conducted to study gender differences and inequalities (Cénat et al., 2020) in assessing and inculcating grit among employees to provide an understanding of organisations in the collectivist culture impacted by sociocultural, economic factors that may affect the grit levels of employees and how these factors can be dealt with, to provide equal opportunities for all individuals to learn, grow and develop their talents as well as adaptability to situations, perseverance of effort, spirited initiative and steadfastness in adverse situations (Singh and Chukkali, 2021).

## Conclusion

The present study pioneers in the research on studying the factors predicting grit, as well as the consequences of grit, from a transpersonal psychology lens and to offer a unique conceptual framework on the same. Grit is crucial for the personal and professional development of employees and others across the organisational sector. Previous research has focused on the importance of grit, but have not recognised the essential factors that can shape grit among individuals at work, from a higher order perspective, such as transpersonal psychology. The study has provided a conceptual understanding of the antecedents of grit, namely, metacognition, flow, optimism and empathy, which were found to have a positive association with each other, and the consequences of grit, namely, job satisfaction and task performance, contextual performance, counterproductive behaviour. Data analyses using path analysis revealed goodness of fit of the measurement models, thereby, substantiating the hypothesis of the study that the transpersonal factors can predict grit, which in turn can result in job satisfaction and job performance. The study offers a beneficial perspective on grit, by predominantly investigating grit from a transpersonal lens, amalgamating useful constructs from positive psychology and examining the consequences of grit inclusive of organisational psychology, from a collectivist setting.

## Author’s Note

The Job Satisfaction Survey developed by Paul Spector in 1985 reserves its copyright permissions (Copyright Paul E. Spector 1994, All rights reserved). The permission for using the Multidimensional Scale of Grit, Flow State Scale, Individual Work Performance Questionnaire and Job Satisfaction Survey was obtained from the authors.

## Data Availability Statement

The raw data supporting the conclusions of this article will be made available by the authors, without undue reservation.

## Ethics Statement

The studies involving human participants were reviewed and approved by the Institutional Ethics Committee of Christ (Deemed to be University), Delhi NCR, India. The patients/participants provided their written informed consent to participate in this study.

## Author Contributions

DA, SC, and SS contributed in the conceptualisation of the research, data analysis, interpretation of data, and manuscript preparation. DA contributed in data collection and in drafting of the paper. All authors contributed to the article and approved the submitted version.

## Conflict of Interest

The authors declare that the research was conducted in the absence of any commercial or financial relationships that could be construed as a potential conflict of interest.

## Publisher’s Note

All claims expressed in this article are solely those of the authors and do not necessarily represent those of their affiliated organizations, or those of the publisher, the editors and the reviewers. Any product that may be evaluated in this article, or claim that may be made by its manufacturer, is not guaranteed or endorsed by the publisher.
